# HIV testing in primary care in the West of Ireland: a mixed-method survey between 2013 and 2023

**DOI:** 10.3399/BJGPO.2025.0034

**Published:** 2025-12-19

**Authors:** Bearach Reynolds, Ruth Naughton, Niamh Lynn, Orlaith Finucane, Maureen E Kelly, Genevieve McGuire, Anne Marie Regan, Fiona Murphy, Helen Tuite, Catherine Fleming

**Affiliations:** 1 Mater Hospital Catherine McAuley Education & Research Centre, Infectious Diseases Department, Mater Hospital, Dublin, Republic of Ireland; 2 Galway Family Planning Clinic, Galway, Republic of Ireland; 3 Consultant GU/HIV Medicine and PrEP Clinical Lead Our Lady of Lourdes, Drogheda, Republic of Ireland; 4 Department of Infectious Diseases, Galway University Hospital, Galway, Republic of Ireland; 5 Discipline of General Practice, School of Medicine, Clinical Science Institute, University of Galway, Galway, Republic of Ireland; 6 Association of University Departments of General Practice in Ireland, Galway, Republic of Ireland; 7 GP and Retired Programme Director Western Training Programme in GP, Clare, Republic of Ireland; 8 ICGP and Psychological Society of Ireland, Dublin, Republic of Ireland; 9 Consultant Infectious Diseases Physician, University Hospital Galway, Galway, Ireland; 10 School of Medicine, University of Galway and University Hospital Galway, Galway, Republic of Ireland

**Keywords:** HIV, primary health care, public health, general practitioners

## Abstract

**Background:**

The year 2022 saw the largest number of human immunodeficiency virus (HIV) cases diagnosed in the Republic of Ireland on record, with a 122% increase on 2021 and a 68% increase compared with the pre-pandemic year of 2019. Late-stage diagnoses and difficulties accessing testing are more common outside of Dublin.

**Aim:**

To assess practices and attitudes to testing in general practice in the West of Ireland.

**Design & setting:**

This cross-sectional mixed-methods study was carried out through University Hospital Galway, which provides all HIV care for the West and North-West of Ireland.

**Method:**

A postal survey was sent to GPs. The survey was based on a study in 2013.

**Results:**

There was a 21.4% response rate (*n* = 136) to the survey, which found 79.4% reported a registered patient living with HIV. Sexual history and patient request were the most common indications for testing and 19.1% used guidelines for testing. More responders had patients living with HIV in 2023 than in 2013 (79.4% versus 59.2%). In 2013, urban GPs were significantly more likely to have sent a test compared with their rural colleagues (*P* = 0.005). This difference was not observed in 2023. Qualitative themes identified included low perceived impact of HIV on daily practice. A key theme was a desire for national guidelines.

**Conclusion:**

GPs report a positive attitude to HIV testing but have concerns regarding undertesting. Urban and rural GPs reported different testing practices but this may be lessening over time. Guideline usage was low. We believe this study highlights areas to improve testing in this region.

## How this fits in

There are limited data on GPs’ human immunodeficiency virus (HIV) testing practices in the West and North-West of Ireland. Our research demonstrates that while GPs report a low impact of HIV on day-to-day practice, they are open to testing. There are concerns regarding workload and missed cases. HIV testing and having a registered patient living with HIV appear more common in 2023 than 2013. While there are reported differences between urban, mixed, and rural GPs, these may be decreasing over time.

## Introduction

The Joint United Nations Programme on HIV/AIDS (UNAIDS) has set goals for human immunodeficiency virus (HIV) testing, treatment, and suppression rates to reach 95% of the eligible population by 2025.^1^ The UK Department of Health and Social Care’s action plan has clearly stated its commitment to achieving zero new HIV infections, acquired immune deficiency syndrome (AIDS) diagnoses, and HIV-related deaths in England by 2030. This strategy places a clear emphasis on scaling up HIV testing across the country.^2^

In the Republic of Ireland, there were 404 new diagnoses of HIV in 2021.^3^ This number has increased significantly with 891 cases diagnosed in 2022, representing a 122% increase on the previous year and a 68% increase on the pre-pandemic level of 2019.^4^ It is notable that a majority of these cases are in those who have migrated to the Republic of Ireland with a known diagnosis of HIV. Living outside of Health Service Executive regions that include Dublin is associated with later-stage diagnoses in consecutive reports from the Health Protection Surveillance Centre (HPSC).^3,4^ There is currently no routine data on HIV testing in primary care in the Republic of Ireland.^3,4^

In international research, living outside of a capital city is associated with slower rates of decline in new HIV diagnoses.^5^ Anxiety levels, lack of time, concerns regarding demands on patient time, increasing age of patient, and patient acceptance have all been cited as barriers to physicians requesting an HIV test in general practice.^^6–9^^

HIV testing has been shown to be acceptable and feasible in non-traditional settings (such as primary care) for both providers and patients.^10^ In an Irish primary care context, opt-out HIV testing has been shown to be acceptable with an uptake of 89.5% among patients attending for routine blood tests in Dublin.^11^ Opt-out testing is endorsed by the Centers for Disease Control and Prevention (CDC) as its preferred method of testing^12^ and its effectiveness in identifying new cases has been highlighted by the recent success of the NHS opt-out programme in emergency departments.^13^

There are no specific guidelines for HIV testing in the Republic of Ireland. Owing to the similarity of population, the most applicable guidelines for use in the Irish primary care context are the British Association for Sexual Health and HIV (BASHH) guidelines on HIV published in 2020.^14^

A survey was carried out in 2013 through the infectious diseases (ID) department at University Hospital Galway (UHG) examining GPs’ HIV-related knowledge and attitudes.^15^ This current research builds on that work and aims to explore the testing practices used by GPs, in a setting outside of the country’s capital city in 2023.

## Method

### Setting

This cross-sectional mixed-methods study was carried out through the ID department in UHG. UHG is a tertiary referral centre and serves a catchment area of approximately one million people in the West and North-West region of Ireland. This area includes small urban centres and a large area of rural communities. UHG provides all HIV care in this region.^16^

### Survey

A postal survey of GPs was carried out in 2013 examining the knowledge and attitudes to HIV in general practice. This survey was created by a group of HIV physicians and GPs and results were presented internationally.^15^ The survey and its results have been included in this report, with consent from the authors.

The current survey has been updated to reflect guidance from the BASHH testing guidelines published in 2020.^14^

The survey is divided into two main sections. There are 22 questions in total.

Questions 1–17 ask GPs to select the most appropriate response(s). These questions produce quantitative data and relate to the GPs’ setting, testing practices, and familiarity with key concepts in HIV.

Questions 17–22 allows for GPs to write in responses and therefore create a qualitative narrative.

The survey asks five open-ended questions related to the GPs’ attitudes and experiences with HIV testing.

The survey was trialled on two independent GPs, and both completed the survey in under 8 minutes. A time requirement of 10 minutes was stated in the invitation letter. No changes to the survey or this procedure were recommended by either GP.

This survey was posted to GPs working in the west of Ireland. This group was defined as those working in the counties of Connacht (Mayo, Sligo, Leitrim, Galway, Roscommon) and Donegal; an area representative of UHG’s catchment area. This list was compiled from the Health Service Executive registry of GPs.^17^ All individual named GPs in County Galway were included. Owing to time and cost constraints, one survey was sent to every GP practice in the other counties, but not every individual GP was contacted.

Surveys were posted between the 26 June and 7 July 2023. All responses were collected and analysed up to the 20 August 2023.

### Analysis

Descriptive statistics were used to analyse the survey results. Subgroup analysis was performed comparing responses from urban, mixed, and rural GPs. Stata (verson 16)^18^ was used to analyse the collected data. Correlation between variables was assessed using the Fisher Exact test, with a *P*-value of <0.05 deemed as significant. Odds ratios (ORs) and confidence intervals (CIs) were calculated comparing urban and rural GP responses.

Further univariate analysis was carried out assessing differences in responses between 2013 and 2023, specifically relating to differences in frequency of reporting of a patient living with HIV and testing for HIV in rural, urban, and mixed settings.

Thematic analysis was used to identify common themes in the answers provided by the responders. Braun and Clarke’s six-phase guide was utilised to perform the thematic analysis.^19^ This involved reviewers becoming familiar with the data and generating initial codes based on commonly cited topics. These codes were then grouped into themes to provide a coherent representation of GPs’ responses.

Results of the 2013 survey were compared with the recent survey to form a comparison of attitudes and barriers with testing among GPs in the region across time.

## Results

### Survey results for 2023

In total, 635 GPs were contacted. There was a 21.4% (*n* = 136) response rate. Of GPs, 31.6% (*n* = 43) self-reported as urban, 22.8% (*n* = 31) as rural, and 45.6% (*n* = 62) as mixed. Further results of the 2023 survey are displayed in Table 1, which shows 79.4% (*n* = 108) reported having a registered patient living with HIV, of which 34.3% (*n* = 37) of GPs diagnosed a patient in their own practice, 25.0% (*n* = 27) were diagnosed in a sexually transmitted infection (STI) clinic, 24.1% (*n* = 26) in secondary care, and 22.2% (*n* = 24) via the migration process. While 98.5% (*n* =134) had tested a patient for HIV in their own practice, 66.4% (*n* = 89) of GPs had tested owing to patient request, 82.1% (*n* = 110) owing to sexual history, 37.3% (*n* = 50) owing to a patient originating from a country of high incidence, and 30.6% (*n* = 41) owing to symptoms of HIV. In addition, 4.4% (*n* = 6) routinely screened new patients for HIV and 24.3% (*n* = 33) believed an HIV test should be sent for every patient attending a doctor at least once. Additionally,19.1% (*n* = 26) reported using guidelines for testing, 80.1% (*n* = 109) were aware of post-exposure prophylaxis (PEP) guidelines, and 77.9% (*n* = 106) were aware of pre-exposure prophylaxis (PrEP) guidelines and its uses. Also, 56.6% (*n* = 77) were familiar with the concept of ‘undetectable = untransmittable’ (U=U).

**Table 1.** Responses to the 2023 survey (*n* = 136)

	Yes (*n*)	No (*n*)	Yes (%)
1) Are any patients in your practice living with HIV?	108	28	79.4%
2) If yes, how were they diagnosed? (Participant may select multiple options as may have more than one registered patient)			
In my practice	37	71	34.3%
In another GP practice	14	94	13.0%
STI clinic	27	81	25.0%
Antenatal screening	10	98	9.3%
Secondary care other than antenatal	26	82	24.1%
Migration pathway	24	84	22.2%
Unknown	37	71	34.3%
3) Do you document their diagnosis as HIV?	101	7	93.5%
4) Do you code their diagnosis as HIV?	82	26	75.9%
5) Have you ever tested a patient for HIV in your practice?	134	2	98.5%
6) If yes, what was the indication for testing? (Select all that apply)			
Patient request	89	45	66.4%
Insurance medical	29	105	21.6%
Needlestick injury	63	71	47.0%
Symptoms of HIV	41	93	30.6%
Screening of persons with occupational risk	37	97	27.6%
Where indicated by sexual history	110	24	82.1%
Person known to be from a country of high incidence	50	84	37.3%
7) Do you test new patients for HIV routinely?	6	130	4.4%
8) In your opinion, should HIV testing be offered to (tick all that apply)			
Every patient attending a doctor at least once	33	103	24.3%
In those with a disclosed risk	114	22	83.8%
Those with possible signs or symptoms of HIV	105	31	77.2%
Patients attending for STI services rather than GP	81	55	59.6%
9) Do you use any guidelines in testing for or managing HIV?	26	110	19.1%
10) Are you aware of PEP guidelines?	109	27	80.1%
11) Are you aware of PrEP and its uses?	106	30	77.9%
12) Are you familiar with the concept of (U=U)?	77	59	56.6%
13) Are you familiar with the concept of HIV indicator conditions?	75	61	55.1%

HIV = human immunodeficiency virus. PEP = post-exposure prophylaxis. PrEP = pre-exposure prophylaxis. STI = sexually transmitted infection. U=U = undetectable = untransmittable

### Comparison of urban and rural GPs 2023

Of responders, 31.6% (*n* = 43) self-reported as urban, 45.6% (*n* = 62) as mixed, and 22.8% (*n* = 31) as rural. Responses of urban, mixed, rural GPs’ responses were compared and full results can be seen in the appendix. Further analysis was performed comparing urban and rural GPs and the results are displayed in Table 2. There were no statistically significant differences between the percentage of rural and urban GPs who reported having a registered patient living with HIV (OR 2.14, 95% CI = 0.65 to 6.97) and whether they had tested a patient for HIV (OR 4.28, 95% CI = 0.16 to 108.59). Urban GPs were more likely to have sent an HIV test based on patient request (OR 5.78, 95% CI = 1.89 to 17.60), symptoms of HIV (OR 5.65, 95% CI = 1.68 to 18.97), and based on the person’s country of origin (OR 11.39, 95% CI = 3.36 to 38.52).

**Table 2.** Comparison of urban and rural GP responses 2023

Questions	Urban Yes (%) *n* = 43	Rural Yes (%) *n* = 31	OR	CI	*P* value
Are any patients in your practice living with HIV?	86.0%	74.2%	2.14	0.65 to 6.97	0.205
Have you ever tested a patient for HIV in your practice?	99.9%	96.8%	4.28	0.16 to 108.59	0.881
Have you sent a test based on a patient request?	86.0%	51.6%	5.78	1.89 to 17.60	0.002*
Have you sent a test based on symptoms of HIV?	46.5%	12.9%	5.65	1.68 to 18.97	0.005*
Have you sent a test as indicated by sexual history?	90.7%	71.0%	3.99	1.09 to 14.46	0.035*
Have you sent a test based on origin from a country of high incidence?	62.8%	12.9%	11.39	3.36 to 38.52	<0.001*
Do you use any guidelines in testing for or managing HIV?	34.9%	6.5%	7.77	1.62 to 37.11	0.01*
Are you aware of PrEP and its uses?	88.4%	71.0%	5.18	1.26 to 21.18	0.022*
Are you familiar with the concept of ‘undetectable = untransmittable’ (U=U)?	79.1%	48.4%	3.63	1.33 to 9.82	0.011*

HIV = human immunodeficiency virus

Bold and asterisk(*) indicates statistical significance.

Urban GPs were also more likely to use guidelines when testing for HIV (OR 7.77, 95% CI = 1.62 to 37.11), be aware of PrEP (OR 5.18, 95% CI = 1.26 to 21.18), and the concept of U=U (OR 3.63, 95% CI = 1.33 9.82).

### Survey results for 2013

Results and basic analysis were provided by the researchers involved in the 2013 survey.

The response rate was 45.6% (*n* = 120). Regarding work setting, 32.5% (*n* = 39) of responders were working in an urban setting, 32.5% (*n* = 39) rural, and 35.0% (*n* = 42) reported working in mixed practices.

The survey found 59.2% (*n* = 71) of GPs reported having a patient living with HIV registered with their practice and 92.5% (*n* = 111) of responders reported testing for HIV. While 43.7% (*n* = 31) of the patients living with HIV were diagnosed in their own GP practice, 35.2% (*n* = 25) were diagnosed in an STI clinic, 29.6% (*n* = 21) were diagnosed by a different GP, 11.3% (*n* = 8) were diagnosed at an antenatal clinic, and 32.4% (*n* = 23) were diagnosed by other secondary care services.

Regarding patients living with HIV, 66.7% (*n* = 26) of urban GPs, 53.8% (*n* = 21) of rural GPs, and 54.8% (*n* = 23) of GPs in mixed settings reported having a patient living with HIV registered with their practice. There was no statistical difference between these groups in this regard.

Urban GPs were significantly more likely to have tested a patient for HIV (*P* = 0.005) than their rural-setting colleagues: 97.4% (*n* = 38) of urban doctors have tested for HIV compared with 79.5% (*n* = 31) of rural doctors, while 92.9% (*n* = 39) of GPs in a mixed setting have tested for HIV. Patient request (74.8%, *n* = 83) and sexual history (73.9%, *n* = 82) were the most cited indications for performing an HIV test. In addition, 15.8% (*n* = 19) of GPs reported using guidelines for the testing for and managing of HIV, while 65.8% (*n* = 79) are aware of post-exposure prophylaxis (PEP) guidelines.

### Comparison of 2013 and 2023 results

Comparison between 2013 and 2023 results are demonstrated in Table 3. Although a larger geographical area was surveyed in 2023, there was no statistically significant difference between the proportion of urban, mixed, and rural GP responders.

**Table 3.** Comparison of 2013 and 2023 results

	2013	2023	*P* value
**Response rate**	45.6% (120)	21.4% (136)	<0.001*
	Yes % (*n*)		
Urban	32.5 (39)	31.6% (43)	0.894
Mixed	35.0% (42)	45.6% (62)	0.098
Rural	32.5% (39)	22.8% (31)	0.093
Are any patients in your practice living with HIV?	59.2% (71)	79.4% (108)	<0.001*
How were they diagnosed?			
In my practice	43.7% (31)	34.3% (37)	0.213
In another GP practice	29.6% (21)	12.9% (14)	0.007*
STI clinic	35.2% (25)	25.0% (27)	0.178
Antenatal screening	11.3% (8)	9.3% (10)	0.80
Secondary care other than antenatal	32.4 (23)	24.1% (26)	0.235
Have you ever tested a patient for HIV in your practice?	92.5% (111)	98.5% (134)	0.027*
Do you use any guidelines in testing for or managing HIV?	15.8% (19)	19.1% (26)	0.515
Are you aware of PEP guidelines?	65.8% (79)	80.1% (109)	0.011*

HIV = human immunodeficiency virus. PEP = post-exposure prophylaxis. STI = sexually transmitted infection. U=U = undetectable = untransmittable

Bold and asterisk(*) indicates statistical significance.

The response rate was significantly higher in 2013 compared with 2023 (45.6% versus 21.4%, *P*<0.001). Significantly more GPs had a patient living with HIV registered in their practice in 2023 (79.4% versus 59.2%, *P*<0.001) and significantly more GPs reported sending an HIV test in 2023 than in 2013 (98.5% versus 92.5%, *P* = 0.027).

Table 4 demonstrates results comparing urban, mixed, and rural GPs between the two years. In 2023, a higher percentage of GPs in each setting reported a registered patient living with HIV. There was a significant increase between the proportion of rural GPs reporting to have tested a patient for HIV in 2023 compared with 2013 (96.8% versus 79.5%, *P* = 0.038). This difference was not observed in urban or mixed GPs.

**Table 4.** Comparison of urban, mixed, and rural practices between 2013 and 2023

	Urban % yes (*n*)			Mixed % yes (*n*)			Rural % yes (*n*)		
	2013 (*n* = 39)	2023 (*n* = 43)	*P* value	2013 (*n* = 42)	2023 (*n* = 62)	*P* value	2013 (*n* = 39)	2023 (*n* = 31)	*P* value
Are any patients attending your practice living with HIV?	66.7% (26)	86.0% (37)	0.065	54.8% (23)	74.2% (46)	0.057	53.8% (21)	74.2% (23)	0.089
Have you ever tested a patient for HIV in your practice?	97.4% (38)	100% (43)	0.475	92.9% (39)	98.4% (61)	0.301	79.5% (31)	96.8% (30)	0.038*

Bold and asterisk(*) indicates statistical significance.

### Qualitative results

Thematic analysis was performed on the qualitative data collected in the 2023 survey. After following the Braun and Clarke steps for thematic analysis, ^19^ seven key themes emerged, which are discussed below.

#### Low perceived risk or prevalence

GPs discussed their perceptions on how HIV impacted on their work. It was commonly felt that HIV had a low impact on their daily work:

*‘Does not feature. Perceived low risk population.’* (GP2)

*‘Relatively low — older, settled practice but obviously no defence!’* (GP4)

#### Willingness to test

It was clear that while GPs reported a reasonably low impact on their workload, there was a general sense that testing was part of their normal working role:

*‘I regularly do HIV testing; it is part of my day-to-day practice.’* (GP5)

*‘Same as any other test, should be done in general practice if there is a possibility of HIV in the differential or if the person requests it or is at risk.’* (GP105)

Positive responses towards GPs testing were common and no responder voiced the opinion that HIV testing was not in the remit of the GP.

#### Concern regarding undertesting

As well as a willingness to test, a common theme noted during the analysis was concern from GPs that they were undertesting:

*‘Should probably be performing more routinely — not sure how much is being missed.’* (GP26)

*‘I probably don’t do enough. I am pro-testing. It is an important diagnosis to make as treatment makes a huge difference to transmission and for the individual affected*.’ (GP55)

Certain GPs highlighted that they were concerned they were undertesting, specifically in those who might come from countries with higher rates of HIV to the Republic of Ireland:

*‘I feel that I should do more, but time is an issue as it takes a while to explain and counsel regarding routine testing based on geographical origin. I do it more routinely for certain communities in case of symptoms.*’ (GP58)

#### Indications for testing

With respect to indications for testing, responses generally highlighted sexual health screening and recurrent infections:

*‘I routinely test for HIV in persons considered to be at risk due to migration from highly endemic areas, when screening for STIs and if not previously done on routine blood tests*.’ (GP100)

*‘Always a consideration, especially as treatment allows people to live normal life expectancy.’* (GP106)

#### Desire for national guidelines or policy

GPs often highlighted the importance of HIV testing and their anxiety around missed cases. Coupled with this, there was a clear perception that testing policies should be standardised or related to national guidelines:

*‘It would be great to give clear “Irish” guidelines about screening for HIV according to current epidemiological situation.’* (GP118)

*‘I don't think we offer it to most patients; I am not familiar with guidelines.’* (GP39)

The role of public health was also highlighted especially around public awareness with an aim to normalise testing:

*‘Both a campaign so that patients are already aware of what HIV is and testing indication guidelines for GPs.’* (GP71)

*‘More public health announcements, people unaware how high numbers are and rising, normalise getting tested.’* (GP81)

#### Logistical barriers to testing

Despite voicing their willingness to test, GPs highlighted the considerable time and cost pressures they are facing as barriers to increased testing and screening:

*‘I agree that it is an important issue, and we perhaps should do better, however general practice has thousands of competing issues, all of which are important, it’s hard to cover everything*.’ (GP78)

There was also an important emphasis put on the cost of sending blood tests in primary care. GPs highlighted that testing involved additional costs and workload for their practice:

*‘Doing bloods for free of charge for medical card patients — lots of work created for practice nurses/GPs following up results after hours — should be properly remunerated*.’ (GP33)

Some GPs highlighted the potential for cultural or language barriers impacting on the likelihood to test:

*‘Time. Language barrier. I find it more difficult with elderly patients. I am not confident bringing it up if I do not feel the patient will understand my explanations due to cultural or language barriers*.’ (GP71)

#### Stigma and fear of causing offence

Participants expressed the view that addressing HIV could have the potential to damage the doctor–patient relationship. This was driven by the concern of offending the patient:

*‘Reluctance on behalf of patient to have a screen, can prompt a much wider discussion about sexuality/family impact that they may not have previously discussed*.’ (GP131)

*‘Patient fear of offense. Asking patients tricky questions when establishing if risk factors present — can be difficult if known them for a long period of time. Patients can get insulted as they may not be expecting a HIV test request so it can require some prompting of why doing it in the first place. Patients feel you are casting aspersions on relationships, orientations etc.*’ (GP4)

*‘We should be doing it a lot more. There is resistance to doing it in older, more established patients due to fear of damaging doctor/patient relationship, but it should be equivalent to getting any test done.*’ (GP4)

## Discussion

### Summary

There are limited data on HIV testing in primary care in the Republic of Ireland. This survey provides an insight into the attitudes and knowledge of GPs in this region. The 2023 results demonstrate that nearly all GPs have tested for HIV and a significant majority have a registered patient living with HIV. Both of these responses appear more universal than in 2013. There are key differences observed between rural, mixed, and urban GPs but our research suggests these differences may be lessening over time. Unlike 2013, we did not find a significant difference between these GP groups in relation to HIV testing or having a patient living with HIV registered in their practice. Notably, rural GPs were significantly more likely to have tested for HIV in 2023 than 2013 (*P* = 0.038). A key finding was the desire for succinct guidelines to optimise testing while allowing GPs to continue on with a considerable and varied workload.

### Strengths and limitations

There are notable strengths to this study. First, there are limited data on HIV testing in primary care in the Republic of Ireland. All GP practices in the West and North-West region were invited to participate maximising the sample included while maintaining feasibility. Data are presented for two points across a 10-year period, which allows us to identify potential points for policy development and intervention.

There are limitations to this study. There may be a selection bias in those who engaged with the survey, including the potential that those with an interest in sexual health may be more likely to respond.

The study involves a relatively small sample size. These small numbers had an impact on statistical analysis, specifically resulting in wide CIs. Nonetheless, we believe the results give us an important insight into HIV testing in primary care.

### Comparison with existing literature

First, we will discuss the different findings between 2013 and 2023. There were more counties included in the 2023 survey. The response rate was lower in 2023 compared with 2013 (21.4% versus 45.6%). It has been noted that response rates from GPs are often less than 30%, lower than the general public and may be falling over time.^^20^^

The breakdown between urban, mixed, and rural was broadly similar between the 2 years. More GPs reported having registered patients living with HIV in 2023 (79.4% versus 59.2%). This finding may be consistent with increasing overall incidence in the Republic of Ireland.^4^ While more GPs reported sending HIV tests in 2023 than 2013 (98.5% versus 92.5%), there is no national published data to support this finding.^3,4^

In 2013, GPs working in urban settings were significantly more likely to test for HIV than their rural or mixed-setting colleagues (*P* = 0.005) . This difference was not observed in the 2023 cohort. In 2013, 97.4% of urban doctors had tested for HIV compared with only 79.5% of rural doctors. Again, we did not find this difference in the 2023 study, with close to 100% of both groups having sent an HIV test. These changes may suggest that testing is more universal across population density in 2023.

While there was a higher percentage of GPs reporting having a registered patient living with HIV across all settings in 2023, this did not meet statisical significance. With regard to testing there was a statistically significant increase in the percentage of rural GPs reporting to have tested for HIV in 2023 compared with 2013 (79.5% versus 96.8%, *P* = 0.038) This may suggest increased awareness among rural GPs in 2023.

In both surveys, sexual history and patient request were the most common indications for testing. Guidelines use was less than 20% in both studies. This may highlight an area for future intervention in this setting.

While a larger geographical area was surveyed in 2023, a similar proportion of urban, mixed, and rural practices allow for comparison and the results may be indicative of some important changes in HIV testing practices in primary care.

Commonly cited physician barriers to testing in this survey included fear of causing offence to the patient along with time and cost restraints. These findings are consistent with international research demonstrating insufficient time, fears of causing offence, and perceived low risk populations as commonly reported barriers.^7,9,21^

The high uptake of bloodborne virus screening (89.5%) in Dublin GP practices indicates that these fears may be out of proportion with patients’ views.^11^ Furthermore, opt-out HIV testing proved acceptable in an urban emergency department in Dublin.^22^While these studies took place in the Republic of Ireland, the demographics differ from our region. An opt-out feasibility study in an acute medical unit in Galway achieved a 40.4% uptake rate when blood-borne virus screening was piloted.^23^ This study compared its rate with the 89.5% rate achieved in the Dublin GP study; concluding that primary care in this part of the country may be a more acceptable location for testing.

A UK study published in 2010, found that while patients generally thought HIV testing to be acceptable, significant negative attitudes were encountered from clinical and administrative staff.^24^ This prompts the question whether it is healthcare workers’ attitudes to testing, rather than patient acceptability that is affecting testing rates.

Urban GPs were significantly more likely to report guideline use, with 34.9% reporting guidelines use versus 6.5% of rural GPs. They were also likely to test based on certain factors such as sexual history (OR 3.99, 95% CI = 1.09 to 14.46) and migration from high-incidence settings (OR 11.39, 95% CI = 3.36 to 38.52). Urban GPs were also more familiar with PrEP and *U*=U. It is unclear from our findings whether setting alone influences these differences or indeed whether guideline use has influenced testing practices.

In both surveys, patient request was cited as the most common indication for testing. This reliance on patient request as an indication for testing has also been noted in international studies.^6^ It has been shown that patients recently diagnosed with HIV would have had a test earlier if they had understood they were at higher risk.^25^ A 2019 study reported more than one-third of gay, bisexual, and men who have sex with men in the Republic of Ireland had not been tested for HIV.^26^ Those who were untested were more likely to live outside Dublin, have the least knowledge about HIV, and be the least confident in accessing an HIV test. This group also reported they would prefer to be tested by their GP than an STI clinic^)^. While high frequency of testing based on patient request may represent an empowered patient cohort, it is a concern that those who are unaware of, or apprehensive to disclose a potential risk will be missed.

The CDC recommends all patients between the ages of 13 years and 64 years get tested for HIV at least once as part of routine health care.^12^ BASHH guidelines recommend universal testing of all attendees at time of phlebotomy where there is extremely high incidence (>5/1000).^14^ While our setting is not of high incidence, only 24.3% believed every patient should be screened at least once suggesting that CDC guidance would not be supported in this region.

Irish GPs face a considerable workload. A 2020 study found that the mean duration of a GP’s day was 9.9 hours and in total 25.4% of work was recorded outside the hours of 9 am–5 pm.^27^ A recent submission to the Irish government from the Irish College of General Practitioners highlighted *‘the workload and workforce crisis in GP*’.^28^

Discussing work pressure of primary care is beyond the scope of this article; however, it is clear from our responders that if additional services are to be expected from GPs, then they must be properly and specifically resourced.

### Implications for research and practice

We have identified several areas for further research. These include exploring the reliance on patient requests for HIV testing and whether this leads to missed opportunities to test. We believe further research could explore whether patients, specifically outside of the urban setting, would be offended if offered HIV testing and/or are staff attitudes more of a determining factor. And finally, if there was introduction of country-specific guidelines whether these would have an impact on testing practices.

In conclusion, GPs are a key part of HIV testing but have concerns regarding undertesting and considerable work pressures in general practice. Notably, rural GPs were significantly more likely to have tested for HIV in 2023 than 2013. While HIV testing appeared more widespread in 2023 than 2013, we found persistent differences between urban and rural GPs. There was a clear desire for guidelines in the 2023 survey responders. We believe primary care in this region could be a key target for policymakers looking to increase testing coverage.

## Data

The dataset relied on in this article is available from the corresponding author on reasonable request.
